# Efficient Photodynamic Therapy on Human Retinoblastoma Cell Lines

**DOI:** 10.1371/journal.pone.0087453

**Published:** 2014-01-31

**Authors:** Jan Walther, Stanislas Schastak, Sladjana Dukic-Stefanovic, Peter Wiedemann, Jochen Neuhaus, Thomas Claudepierre

**Affiliations:** 1 Department of Ophthalmology, Faculty of Medicine, University of Leipzig, Leipzig, Germany; 2 Department of Urology, University of Leipzig, Leipzig, Germany; Université de Technologie de Compiègne, France

## Abstract

Photodynamic therapy (PDT) has shown to be a promising technique to treat various forms of malignant neoplasia. The photodynamic eradication of the tumor cells is achieved by applying a photosensitizer either locally or systemically and following local activation through irradiation of the tumor mass with light of a specific wavelength after a certain time of incubation. Due to preferential accumulation of the photosensitizer in tumor cells, this procedure allows a selective inactivation of the malignant tumor while sparing the surrounding tissue to the greatest extent. These features and requirements make the PDT an attractive therapeutic option for the treatment of retinoblastoma, especially when surgical enucleation is a curative option. This extreme solution is still in use in case of tumours that are resistant to conventional chemotherapy or handled too late due to poor access to medical care in less advanced country. In this study we initially conducted in-vitro investigations of the new cationic water-soluble photo sensitizer tetrahydroporphyrin-tetratosylat (THPTS) regarding its photodynamic effect on human Rb-1 and Y79 retinoblastoma cells. We were able to show, that neither the incubation with THPTS without following illumination, nor the sole illumination showed a considerable effect on the proliferation of the retinoblastoma cells, whereas the incubation with THPTS combined with following illumination led to a maximal cytotoxic effect on the tumor cells. Moreover the phototoxicity was lower in normal primary cells from retinal pigmented epithelium demonstrating a higher phototoxic effect of THPTS in cancer cells than in this normal retinal cell type. The results at hand form an encouraging foundation for further in-vivo studies on the therapeutic potential of this promising photosensitizer for the eyeball and vision preserving as well as potentially curative therapy of retinoblastoma.

## Introduction

Retinoblastoma is a genetically determined tumor due to mutations or deletions of both copies of RB1, a tumor-suppressor gene encoding a 110 kDa nuclear protein involved in the control of neoplastic growth [1]. The retinoblastoma protein (pRb) is responsible for a major G1 checkpoint, blocking S-phase entry by targeting E2F transcription factors [2]. Alterations in the pRb/E2F pathway are commonly found in human cancers, and in the absence of pRb multiple pathways are activated leading to increased tumor growth [3]. Retinoblastoma arises from a mixture of transformed retinal progenitors with Müller- and photoreceptor-like characteristics and bipotential differentiation status [4], [5]. Retinoblastoma is the most common intraocular tumor of infancy and early childhood affecting 1 out of 18.000 births [6]. Most cases develop in the first or second year of life, although later presentation can occur and very rare cases have even been reported in adults [7]. In about 40% of patients, the disease is bilateral denoting a hereditary form of the disease. Absence of treatment is always fatal and patients die of intracranial extension and disseminated disease within 2 years. While the vital prognosis for RB is excellent in industrialized countries with cure rates greater than 95%, in low income countries the survival ranges between 25 to 70% [8]. Chemotherapy is adapted for early tumors but enucleation is still a common strategy in the treatment of retinoblastoma worldwide [9]. The use of this extreme surgical solution is now rare in Western country but still occures due to the development of resistance against chemotherapy agents in advanced retinoblastoma [10]. In addition, complications may arise from the use of radiotherapy and systemic chemotherapy, including cataracts, radiation retinopathy and developpement of secondary malignant neoplasms and leukemia [11]–[13]. In less advanced country, however, ennucleation is still a widely used therapy. This is due to combination of various factors including poor network of paediatrician, limited access to medical center with proper oncology service, cost of treatments... Resistance to current therapeutic approaches against retinoblastoma and lack of easily deployable strategy in less advanced countries underline the need for the development of new and efficient therapies.

A promising treatment for neuro-ophthalmic cancer consists in photodynamic therapy (PDT). Discovered 100 years ago by Hermann von Tappeiner and Oscar Raab [14], PDT requires the simultaneous presence of a photosensitizer, light and oxygen inside the diseased tissue. The photosensitizer accumulates in the target cells and absorbs light at specific wavelengths. The energy is transferred to endogenous oxygen and highly reactive oxygen species (ROS) as well as singlet oxygen are generated. Treatment with appropriate light doses generates ROS and directly leads to cell death without any possible development of a resistance mechanism [15], [16]. As most of the antidrugs, photosensitizers exhibit a higher uptake by cancer vs normal cells, thus allowing a very selective eradication of the malignant tumor while sparing the surrounding tissue [15], [17]. Altogether PDT is rapid, it has no long-term side effects, and most importantly stimulates the immune system, thus maximizing the tumoricidal effect [18]–[20].

Here, we proposed to use as a photosensitizer the tetrahydroporphyrin-tetratosylat (THPTS), a pure, positively charged, water soluble and chemically stable synthetic substance [17]. THPTS accumulates very strongly in the cancer cells compared to healthy cells, with a tumor vs normal tissue ratio (TNTR) ranging from 8 to 50. THPTS possesses no toxic effect in pre-incubated cells without irradiation. Consistent with the fact that positively charged photosensitizers specifically induce the death of tumor cells by apoptosis [21], it has been shown that THPTS-PDT followed by light irradiation induced an efficient apoptosis in the target cells [22]. In addition, skin photosensitivity, a typical side effect of PDT can be neglected as the turn over of the product is high in healthy cells [17]. Also, heat damage to the surrounding tissue is absent as the amount of energy needed for THPTS activation is reduced. THPTS-PDT, in combination with an appropriate laser light at 760 nm, penetrates up to 25 mm of tissue, much deeper than commonly used photosensitizers [22]. The higher penetration depth of THPTS-PDT enables the treatment of tumors with a size of ca. 15 mm which are not within the scope of actual photosensitizers [17]. Altogether THPTS exhibits several advantages compared to the photosensitizers actually used in clinic and may consist in a new anticancer drug to target retinoblastoma cells while sparing the vision.

## Materials and Methods

### Human retinoblastoma cultures

Prior to survival assay and RT-PCR, human retinoblastoma cell lines Y79 (# HTB-18, ATCC, Manassas, VA) and WERI Rb-1 (# HTB-169, ATCC) were maintained in flasks with DMEM Glutamax medium (#21885, Invitrogen) containing 1000 U/ml penicilin and streptavidin (#15140-122, Invitrogen) and 10% fetal calf serum (#16000-044, Invitrogen) at 37°C, 85% humidity and 5% CO2. For immunocytochemistry, Y79 and WERI-Rb-1 cells were cultured on BD Falcon 8 wells glass chambers slides (#354108, Becton-Dickinson, Franklin Lakes, NJ) previously coated with 25 µg/ml of laminin-1 (L2020, Sigma, St. Louis, MO). Cells were seeded at 60.000 cells/cm2 and cultured for a week in DMEM Glutamax medium (#21885, Invitrogen) supplemented with 250 µM dibutyryl cyclic AMP (Sigma) 1000 U/ml penicilin and streptavidin (#15140-122, Invitrogen) and 10% Lipumin (#F11-014, PAA Laboratories, Pasching). As described previously [23], [24], those conditions allowed the adherence of the retinoblastoma cell lines on the glass surface, therefore easing the immunocytochemistry staining process and analysis.

### Primary human retinal pigmented epithelium cell cultures

The use of human material was approved by the Ethics Committee of the University of Leipzig, and all procedures were performed in accordance with the Declaration of Helsinki. All tissues were obtained after receiving written informed consent from the patients or their legal representatives. Copies of the consents were sent to the Ethics Commitee of the University for archiving. Eyes were obtained from adult post-mortem donors without reported eye diseases within 48 h of death. Primary human RPE cells were isolated from the eyes as described previously [25]. The obtained RPE cells were cultured in flasks (Greiner) in Ham F-10 medium containing 10% fetal calf serum, Glutamax II, and penicillin/streptomycin at 37°C in a 5% CO_2_ atmosphere.

### Photodynamic therapy on retinoblastoma and retinal pigmented epithelium cells

For each sample, 500 µl of stock solution of the different cell suspensions (Y79; WERI Rb-1 cell lines and RPE cells) at 10^6^ cells/ml were cultured on 12 well plates (Greiner Bio-One, Flickenhausen Germany). The samples were treated with the respective amount (0, 25 µg/ml; 50 µg/ml; 100 µg/ml; 200 µg/ml) of THPTS made from a stock solution at 10 mg THPTS/ml in water, prepared extemporaneously. All samples were then placed in the incubator for different incubation periodes (0.5 h, 1.5 h, 3 h, 6 h, 12 h, and 24 h). After the administration of THPTS, all samples were handled under a halogen desk lamp equipped with a monochrome filter at 325 nm in order to prevent unintended activation of the photosensitizer. To avoid THPTS to interfere with the proliferation assay we performed three washing steps with PBS. Retinoblastoma and RPE cells (with prior trypinisation) were collected into 1.5 ml tubes and centrifuged at 1000 rpm for 5 min. the supernatant was discarded and the remaining pellet re-dissolved in 500 µl PBS. After the third centrifugation the pellet was re-dissolved in 500 µl fresh THPTS-free culture medium. To ensure an equal distribution of the laser light, 100 µl of cell suspension was then distributed in 96 well plates prior to irradiation.

Except for the dark toxicity assay, all samples were then irradiated with 60 J/cm^2^ at 760 nm using a diode laser (Ceralas D, Ceramoptec GmbH, Bonn, Germany). After incubation for 24 hours, a survival assay using tetrazolium salt wst-1 (#05015944001, Roche, Mannheim Germany) was performed by adding 10 µl of wst-1 reagent to each well. After 2 hours the resulting colorimetric reaction of the formazan dye generation was measured at 450 nm and 650 nm on a microplate reader (Spectramax 250, Molecular Devices, Sunnyvale, CA). The amount of formazan dye formed reflected the number of metabolically active cells in the culture and therefore, the measured absorbance directly correlated to the number of viable cells. Background value obtained for the medium alone was withdrawn from the resulting data.

### Clearance of THPTS

To investigate the dynamic of the compound clearance, 100 µl from the 500 µl of the different cell samples (prepared as described above) were distributed in 96-well plates, irradiated and analyzed after 24 hours of incubation. The remaining 400 µl of each cell samples were transferred into a new 12-wells plate and maintained in the incubator for 30 hours. After this additional incubation, the samples were washed as described above and 100 µl of the different cell suspensions were then transferred onto a 96-well plate, irradiated and analyzed 24 hours later, now leaving 300 µl for another 30 hours to repeat the procedure once again. This allowed us to analyze the changes in THPTS-PDT efficiency after long incubation periods reflecting THPTS clearance from the cells.

### Total RNA extraction and cDNA synthesis

For real-time-PCR analysis, each 500 µl sample of the stock solution of both retinoblastoma cell lines was prepared on 12-well plates as described above. All samples except control were supplied with THPTS at a concentration of 200 µg/ml and incubated for three hours (37°, 5% CO2). After incubation the samples were washed three times and 100 µl were then transferred onto a 96-well plate and irradiated with the laser. All samples were then again returned to the incubator for various periods of time (0.5 h; 1.5 h; 3 h; 4.5 h; 6 h; 12 h; 24 h). After the respective incubation time, 350 µl of RLT-Buffer (Qiagen, Hilden, Germany) were added to each sample to disrupt the cells and total RNA was extracted from Rb-cells by using the RNeasy Mini Kit (Qiagen). The quality of the RNA was analyzed by agarose gel electrophoresis. The A_260_/A_280_ ratio of optical density was measured using the GeneQuantpro device (Pharmacia, Uppsala, Sweden), and was between 2.0 and 2.2 for all RNA samples, indicating sufficient quality. After treatment with DNase I (Roche, Mannheim, Germany), cDNA was synthesized from 1 µg total RNA using the RevertAid H Minus First Strand cDNA Synthesis kit (Fermentas, St. Leon-Roth, Germany).

### Real-time RT-PCR

The relative mRNA levels of the different genes (hATF3; hJUN; hHSP70B; hp27; hGADD45G; hGADD153; hHSP105) in THPTS-PDT-treated retinoblastoma cells were determined in comparison to the levels of the controls (no THPTS-PDT). Quantitative real-time RT-PCR was performed with the Single-Color Real-Time PCR Detection System (BioRad, Munich, Germany) using the following forward/reverse primer pairs sequences (5′-3′; size of the endproduct in bp): hGAPDH, GCAGGGGGGAGCCAAAAGGGT/TGGGTGGCAGTGATGGCATGG (219); hATF3, TGTCCATCACAAAAGCCGA0GGTAGC/CTCCTTCTTCTTGTTTCGGCACT (107); hJUN, GCCAGAGCCCTGTTGC/GAAGGTCGTTTCCATCTTTGC (102); hHSP70B, GTGGGGGCACCTTCGATGTGT/TGGTTCACGAGCCGGTTGT (118); p27/KIP1, AAGCGACCTGCAACCGACGATTCTT/GCTCCACAGAACCGGCATTT (100); hGADD45G, CGAGTCAGCCAAAGTCTTGAACGTG/GAAAGCCTGGATCAGCGTAAA (121); hGADD153, TGTCTTCAGATGAAAATGGGGGTAC/CAGAGAAGCAGGGTCAAGAGT (95); hHSP105, CCCCGTCAGTCATATCATTTGG/TGTTTGCATGAGTGATTTGCTG (81).

The PCR solution contained 1 µl cDNA, specific primer set at 0.2 µM each and 10 µl of a 2× mastermix (QuantiTect SYBR Green PCR Kit; Qiagen) in a final volume of 20 µl. The following conditions were used: initial denaturation and enzyme activation (one cycle at 95°C for 15 min); denaturation, amplification and quantification, 45 cycles at 95°C for 30 s, 58°C for 30 s, and 72°C for 1 min; melting curve, 55°C with the temperature gradually increased (0.5°C) up to 95°C. All PCR data were checked for homogeneity by dissociation curve analysis. Fluorescence changes were monitored after each cycle, *C*t (threshold cycle) values for amplification of the respective mRNA and GAPDH mRNA were defined, and the levels of the different mRNA in each sample were standardized to the endogenous hGAPDH level. Comparable efficiencies for targeted gene and hGAPDH mRNA amplification were determined by analyzing serial cDNA dilutions. The changes in mRNA expression for the respective genes were calculated according to the 2^−ΔΔCT^ method (CT, cycle threshold), with ΔCT  =  CT_treated_− CT_control_ and ΔΔCT  =  ΔCT_target gene_ – ΔCT_control_. The amplified bands were analyzed by standard agarose gel.

### Immunocytochemistry and live recording

After laser illumination, Y79 cells were fixed for 15 minutes in a 4% formaldehyde solution in PBS before immunostaining process. Non specific sites were blocked and cells were permeabilized using a solution of 30% casblock (InVitrogen) and 0.2% Triton X100 in PBS for 30 minutes at RT. Actin filaments were revealed using phalloidin coupled to Alexa 488 (#A12379 InVitrogen) and used at 1/50. DAPI (InVitrogen) nuclear staining was achieved in the last washing steps. Slides were mounted with Fluoromount-G (EMS, Hatfield, USA), observed using a Zeiss Axioplan 2 fluorescent microscope and picture taken with a Axiocam MRc5 digital camera coupled to Axiovison 4.6 software (Carl Zeiss, Jena Germany). Time lapse imaging was achieved using 100 µg/ml THPTS incubated with cells for 1.5 h. After compound activation, images were taken every 30 seconds for 40 minutes using an inverted microscope (Aviovert 25, Carl Zeiss) coupled to a digital camera.

For subcellular localization of THPTS, Y79 cells were incubated for 2 h at 37°C with 200 nM MitoTracker Green FM (M7514, Invitrogen, Karlsruhe, Germany) and 200 µM THPTS. The cells were visualized at a LSM 5 Pascal (ZEISS, Oberkochen, Germany) laser scanning microscope using an Achroplan 63×0.95 water immersion objective. M7514 was excited at 488 nm and emission was detected at 505–530 nm; THPTS was excited at 514 nm and detected using a long pass LP 650 nm filter set. A FRET (Förster Resonance Energy Transfer) system was developed to visualize THPTS using excitation at 488 nm exciting the M7514, which in turn excited nearby THPTS with fluorescent emission at 516 nm. The detection of THPTS was performed with the same LP 650 nm filter set. For oxidative stress visualization, mitochondria were labeled (200 nM, 2h, 37°C) with the chemically reduced forms of the tetramethylrosamine (MitoTracker Orange CM-H_2_TMRos, M7511, Invitrogen) with or without 200 µM THPTS incubation. M7511 is not fluorescent in reduced form. M7511 has an excitation maximum at 560 nm and would emit at 575 nm only if oxidized. To achieve fluorescence separation at best, we excited M7511 at 488 nm (about 10% efficiency) and detected the fluorescence with a band pass filter set (560–615 nm). THPTS was excited at 514 nm (99% efficiency) and detected using a long pass filter set (>650 nm). Though M7511 shows 30% excitation efficiency at 514 nm, the emission above 650 nm is well below 1%, ensuring appropriate separation of the dyes in multi-track laser scanning microscopy.

Oxidation of M7511 was enhanced by irradiation of the cells with UV-light using a DAPI filter set (<300 nm – 420 nm transmission).

### Data analysis

Each experiment was repeated at least 3 times per condition. Bar diagrams display the means of cells survival rate (± SEM). Comparisons between the means across all experimental conditions were made by Welch T-Test. *P* < 0.05 was considered statistically significant.

## Results

### Effect of THPTS-PDT on retinoblastoma cell lines

**Figure 1:**
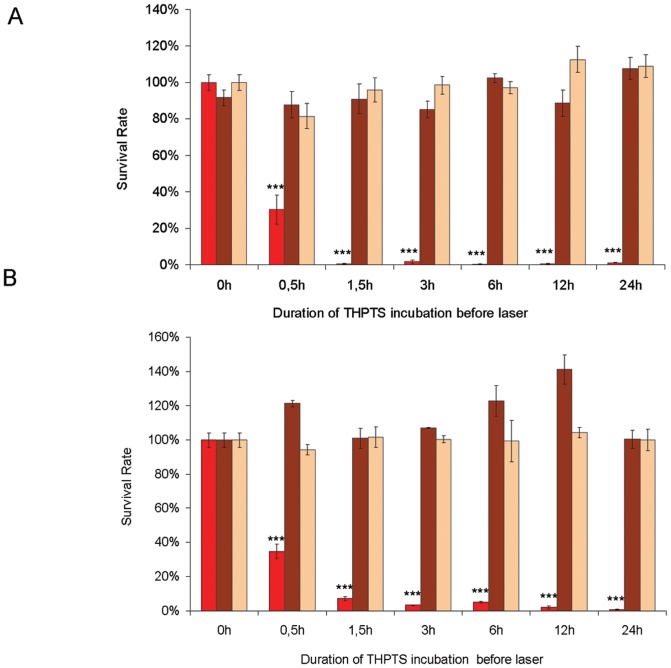
THPTS-PDT on retinoblastoma cell lines. In vitro cell survival analysis of WERI Rb-1 (A) and Y79 (B) cells irradiated with laser after incubation with THPTS (200 µg/ml) for 0.5 h, 1.5 h, 3 h, 6 h, 12 h or 24 h (red bars) showed a minimal survival rate of 0% –5% after an incubation time of 1.5 hours (WERI Rb-1) or 3 hours (Y79). When incubated with THPTS (200µg/ml) without irradiation (dark toxicity) for 0.5 h, 1.5 h, 3 h, 6 h, 12 h or 24 h (brown bars), the survival rate relative to control varied from 85.2% (3 h) to 107.7% (24 h) in WERI Rb-1 and from 100.0% to 141.1% in Y79. Laser irradiation alone (light orange bars) lead to no significant changes of the survival rates relative to controls.

We first incubated the Rb-cell-lines with THPTS for different periods of time (0.5 h, 1.5 h, 3 h, 6 h, 12 h and 24 h) at a concentration of 200 µg/ml in order to investigate the effectiveness of the PDT as well as the dark toxicity of the compound depending on the application time (Figure 1). At different time points the medium was replaced by THPTS-free medium and the corresponding samples were then irradiated with the 760 nm laser. After another 24 hours (at 37°C; 5% CO_2_; darkness) the viability was analyzed with WST-1 assay (Roche, Mannheim Germany). This allowed us to gain information on the dynamics of the uptake of the compound into the cells and to determine the most effective time of incubation with THPTS. We found that there was no measurable dark toxicity within the tested period of time using 200 µg/ml of THPTS. In addition the sole laser irradiation of the cells without THPTS led to no significant changes in the survival rates compared to the controls. Using 200 µg/ml THPTS followed by PDT, demonstrated the cytotoxic effect of the treatment even for the shortest incubation time tested, as the survival dropped below 35% for the two cells lines tested after 0.5 h (30.2 +/–8.0% and 34.9 +/– 4.1% for WERI Rb-1 and Y79 respectively). The samples submitted to THPTS-PDT showed a maximal effect on the cell survival after 1.5 h (WERI Rb-1) and 3 h (Y79) of incubation with the compound, respectively. Beyond these times points, the effectiveness of the induced photo-killing of retinoblastoma cells reached 100% as was no detectable metabolism of the formazan in thoses cells compared to the control situation (p<0.001).

### Dose-dependent and incubation effects of THPTS-PDT

**Figure 2:**
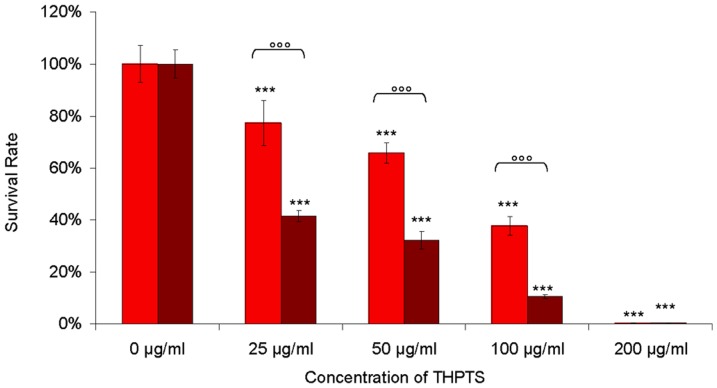
Dose and incubation time effects. The incubation of WERI Rb-1 with THPTS at concentrations of 25 µg/ml, 50 µg/ml, 100 µg/ml or 200 µg/ml for 1.5 h (red bars) lead to survival rates of 77.3%, 65.9%, 37.8% and 0.4%.respectively. An incubation time of 3 h (deep red bars) roughly doubled the cell death rates (at the lower concentrations) with survival as low as 41.5%, 32.2% 10.6% and 0.4% respectively, (*** p<0.001 comparison with control without THPTS; °°° p<0.001 for the comparison between 1.5h and 3h of THPTS incubation)

As a next step we compared the effect of different concentrations (25 µg/ml, 50 µg/ml, 100 µg/ml, 200 µg/ml) of THPTS on the viability of WERI Rb-1 cells (Figure 2). The samples were again incubated with the respective amount of the compound for 1.5 h and 3 h, treated with the laser and then tested for viability using the WST-1 assay. The results show a clear dose-dependent effect of the THPTS-PDT with survival rates of 77.3%, 65.9% 37.7% and 0.4% respectively after 1.5h. At lower concentrations (25, 50 and 100 µg/ml), there was also a significant dependency on the application time (p<0.001 for all time points). When incubated for 3 hours with 25 µg/ml, 50 µg/ml, 100 µg/ml THPTS, the survival was decreased to 41.5%, 32.2% and 10.6% respectively, corresponding to 1.8, 2.0 and 3.5 fold increases of the photo-killing just by doubling the incubation time of the respective THPTS concentration.

### Effect of THPTS-PDT on cell survival in non-malignant retinal cells compared to retinoblastoma

**Figure 3:**
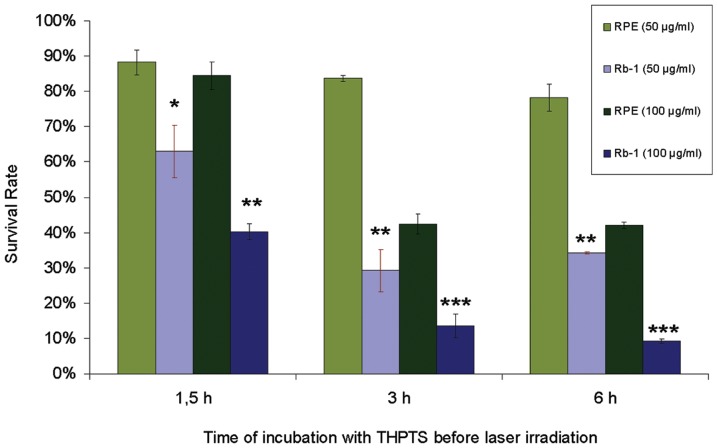
Rate of WERI-rb1 and primary RPE survival following THPTS treatment. As low as 50 µg/ml of THPTS induced an effect on retinoblastoma cell line as survival dropped to 62.9% after 90 min of incubation (light blue bars). In RPE, using the same condition the survival decreased slightly to 88.2% (light green bars). When increasing the THPTS concentration to 100 µg/ml the compound led to a reduced survival to 40.3% in retinoblastoma cell lines (deep blue bars) and only affect slightly the normal primary RPE cells over the same incubation period (84.5%, deep green bars). Increasing the incubation to 3 hours induced a more drastic effect of THPTS even with low concentration. 50 µg/ml of THPTS induced a survival of only 29.2% of the We-Rb1, a rate that dropped to 13.5% when using 100 µg/ml. Meanwhile the primary RPE cells survival is 83.7 and 42.4% with the same conditions. A survival significantly higher than for Rb1. After 6h the survival is not further affected in RPE cells as it was found to be at 78.3 and 42.1 using 50 and 100 µg/ml of THPTS. In Rb cells also the survival was only slightly further affected compared to 3 h of incubation (34.3% and 9.2% respectively). Asterisks indicate significant differences between Rb1 and RPE cells in comparable situations of concentration and incubation length.

To explore the differential cytotoxicity of THPTS-PDT on non-degenerated retinal cell populations, we compared the phototoxic effect in retinoblastoma (WERI Rb-1) and normal primary human retinal pigmented epithelium cells (hRPE). We measured the survival rate of RPE cell treated 3h with the higher dose of THPTS (200 µg/ml) and observed that normal primary cells survived at 32.63 +/– 1.48%, while photokilling of Rb cells with this THPTS concentration and incubation time was maximal. In addition, in absence of laser activation (dark toxicity), RPE survival with those parameters was found to be at 106,99 +/– 7.76%. THPTS had therefore no cytotoxic effect per se on RPE cells in vitro and affected RPE cells to a lower extend when activated by laser. As seen in figures 1 and 2, cell survival can be modulate by changing incubation time and THPTS concentration. In order to allow a high photokilling of tumor cells together with a limited effect on normal cell, we lowered THPTS concentration on Rb and RPE cells and modulated the lenght of incubation with the photosensitizer. We incubated both cell cultures with THPTS at 50 µg/ml and 100 µg/ml for 1.5 h, 3 h and 6 h before we supplied them with fresh THPTS-free medium and administered the laser irradiation (Figure 3). While after 1.5 h of incubation the WERI Rb-1 cells showed survival rates of 62.9% (50 µg/ml) and 40.3% (100 µg/ml), the hRPE still presented 88.2% and 84.5% of survival, respectively. This represents a 1.4 fold (p<0.05) survival increase for RPE vs Rb cells treated with 50 µg/ml of THPTS and 2.1 fold (p<0.01) survival increase when treated with 100 µg/ml THPTS for 90 minutes. The relative difference was even stronger after 3 hours of incubation, as WERI Rb-1 survived to 29.2% and 13.5% and hRPEs to 83.7% and 42.4% when using 50 and 100 µg/ml THPTS respectively. This correspond to a 2.9 (p<0.01) and 3.1 (p<0.001) fold increase of survival to the treatment for hRPE vs Rb with those settings. After 6 hours of incubation the PDT treatment did not further affect the survival, indicating that the maximal effect on these cells with these parameters was reached.

### Turnover of THPTS

**Figure 4:**
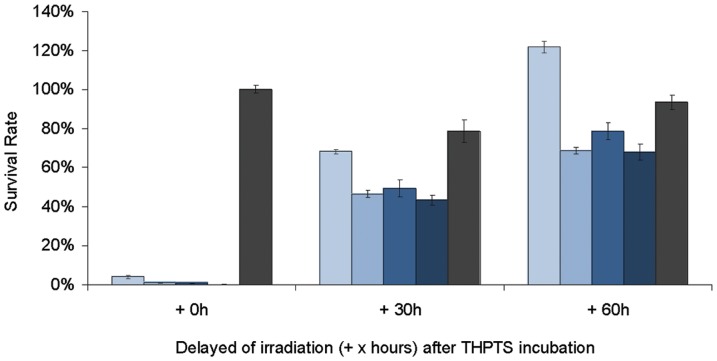
THPTS turn over in cells. Loss of efficiency of THPTS in cells when irradiated after 30 and 60 h post incubation with 200 µg/ml THPTS for 0.5 h (lighter blue bars), 1.5 h (light blue bars), 3 h (blue bars), 6 h (deep blue bars). Survival was low as 0.1 to 4.0% when cells were irradiated right after incubation with the compound (0.5 to 6 h). The survival rate inceased if the time before irradiation was extended to 30h, with low incubation period of 0.5 h, the survival was already back to 68.2% and For all other incubation lengths the survival was found to be between 43.4 to 49.4% after 30 h. The loss of efficiency of THPTS was even greater after 60 h, with a survival of 121.8% for the shortest incubation with THPTS (0.5 h) and ranging from 67.9 to 78.7% for the longer incubations. This represented a reduced efficiency for PDT of 1.79 fold for the shortest incubation and of 1.45, 1.56, 1.59 fold for the long incubation periods (1.5 to 6 h), thus approaching the half-life of the product within living cells. Dark toxicity (grey bars) after 6h of incubation with THPTS was found to be at 100.1% without delay, 78,7% when irradiation was delayed 30h and 93.5% when delayed 60h.

We examined whether THPTS is released by the cells when not being activated by laser light. We incubated the WERI Rb-1 cells with THPTS (200 µg/ml) for 0.5 h, 1.5 h, 3 h and 6 h (Figure 4). We then treated fractions of the respective samples with laser irradiation and then measured the survival rates (time point 1). The survival rates were found between 0% and 4% for all of the four conditions. The remaining fractions of the cultures were supplied with fresh THPTS-free medium. After allowing the cells to release the compound into the medium for about 30 h (time point 2  =  time point 1 + 30h) we again irradiated a fraction of the cell culture and obtained survival rates of 68.2% for the 0.5 h samples and 46.6%, 49.4% and 43.4% for the 1.5, 3, and 6 hours samples, respectively. After repeating the same procedure for another 30 h (time point 3  =  time point 1 + 60 h) we obtained survival rates of 67.9%, 78.7%, 68.7% and 121.8%, respectively, relative to controls. Each washing step resulted in a 500x dilution of the active compound present in the medium. It is therefore unlikely that the release of the compound is affected by an accumulation in the medium and we can consider the clearance of the compound to be unrestricted to the greatest extent. Survival rates reflect the amount of compound still present in the cell. Survival increases observed at time point 3 ranged from 1.8 to 1.5 fold compared to survival values at time point 2, thus reflecting a corresponding loss of efficiency of PDT due to THPTS turnover in Rb cells. Based on these observations and the reported properties of the compound [17], 30 h time point was found close to the half life of THPTS in the cells.

### Gene expression

**Figure 5:**
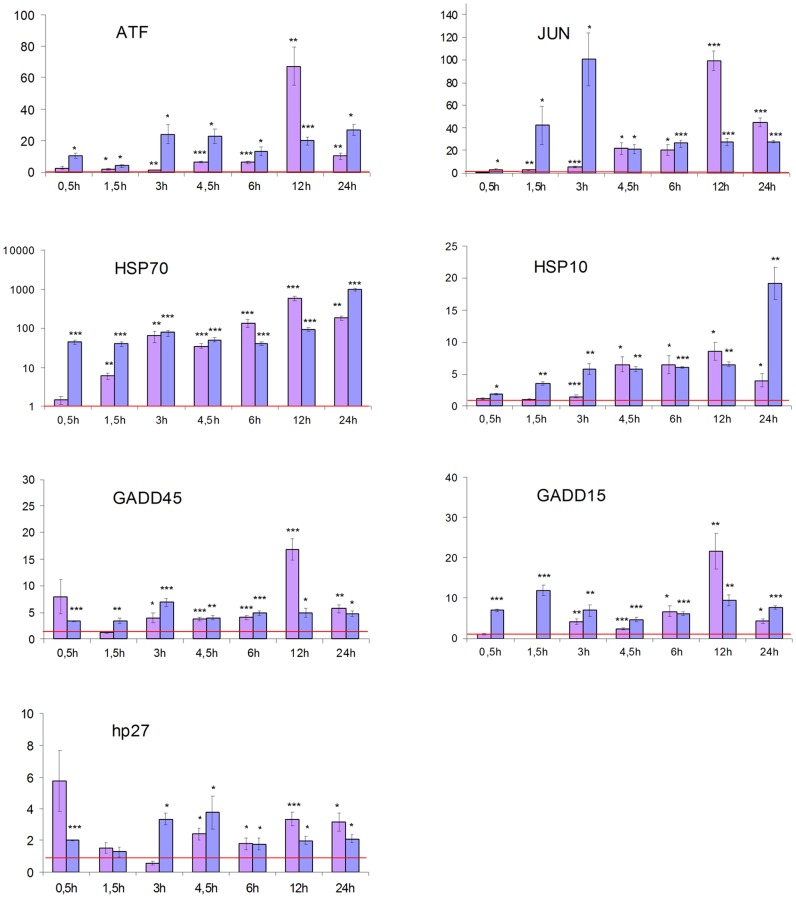
Quantitative gene expression. The expression of genes related to apoptosis showed rapid and significant increases after treatment with THPTS-PDT compared to the controls (depicted as the red line reflecting the expression baseline). Y axis corresponds to the relative expression vs control while x axis corresponds to the different time points (0.5, 1.5, 3, 4.5, 6, 12 and 24h). The pink bars reflect the Y79 and the violet bars the WERI Rb-1 cells. ATF3 showed a significant increase of up to 67.25 fold (p<0.01) in Y79 cells and a 19.87 fold (p<0.001) increase in WERI Rb-1 cells within 12 hours after treatment. Maximal increase for hJUN expression was obtained with values up to 99.30 fold (p<0.001) in Y79 cells and up to 100.60 fold (p<0.05) in WERI-Rb-1, 12 and 3 hours after PDT respectively. hHSP70B was increased significantly after 1.5 and 0.5 hours for Y79(6.09 fold, p<0.01) and WERI Rb-1 (43.84 fold, p<0.001) respectively. With maximal increase values up to 584.24 fold (Y79, p<0.001) and 979.00 fold (WERI Rb-1, p<0.001). hHSP105 levels increased significantly after 3 h and 0,5h in Y79 (1.43 fold, P<0.001) and in WERI Rb-1 cells (1.87 fold, p<0.05) respectively; reaching a maximum of 8.55 fold (Y79, p<0.01) to 19.20 fold (WERI Rb-1, p<0.001) within 24 h. The DNA damage related mRNA levels of hGADD45G (Y79: max. 16.81 fold increase after 12 h, p<0.001; WERI Rb-1: 6.88 fold increase after 3h, p<0.001) as well as hGADD153 (Y79: max. 21.57 fold increase after 12 h, p<0.01; WERI Rb-1: 11.90 fold increase after 1.5h, p<0.001) were significantly increased within the first 4.5 h after PDT for both cell lines. The levels of hp27 were increased significantly increased in both cell lines. The maximum reached a 3.34 fold increase after 12 h (p<0.001) for the Y79 cells and a 3.75 (p<0.05) fold increase for the WERI Rb-1 cells 4.5 h after the treatment. Note that for hHSP70B, y axis is in log scale.

By quantitative RT-PCR (Figure 5), we furthermore determined the effect of THPTS-PDT on the mRNA-levels of genes related to apoptosis (hATF3; hJUN; hHSP70B) or stressful growth arrest (hp27) including DNA damage (hGADD45G; hGADD153) and other severe stress (hHSP105) [26]. We were able to show that the mRNA levels of several genes related to cellular damage and apoptosis were in great parts strikingly increased. In this regard we observed significant (p<0.05 to p<0.001) up-regulation of mRNA levels of ATF3, JUN and HSP70B, demonstrating the activation of apoptotic pathways. The enhancements ranged from 67-fold increases for ATF3 and 99-fold increases for JUN to well above 900-fold increases of HSP70B transcripts which are considered to be specific for Fas-induced apoptosis [27]. The demonstrated increases of gene transcripts related to cellular stress (HSP105) and severe DNA-damage in particular (GADD45G and GADD153) give further evidence of the cellular damage induced by the THPTS-PDT within the retinoblastoma cells. Significant 16- to 21 fold increases were recorded for the maximal relative expression of the three gene transcripts. These findings are confirmed by up to fourfold increases of the hp27 transcripts compared to the controls, indicating growth arrest as a result of the subcellular damage and ongoing apoptotic process [28].

### Immunocytochemistry and live recording

**Figure 6:**
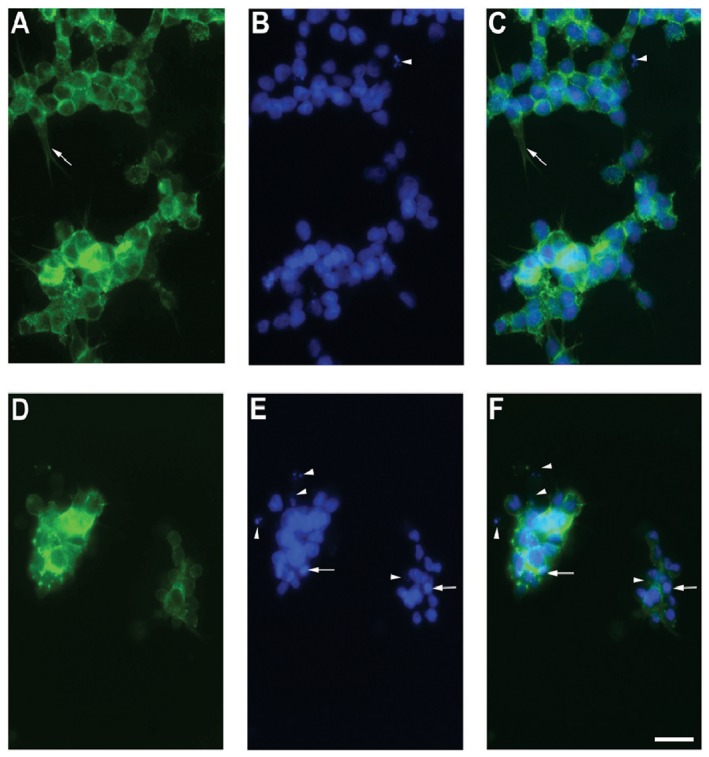
Immunocytochemistry. Y79 cells were cultured in 10% lipumin and incubated with low dose of THPTS (25 µg/ml) for 90 min. After formaldehyde fixation, cells were stained with DAPI nuclear marker and phalloidin coupled to Alexa 488. In control without laser illumination Rb cells showed a strong development of filopodia as seen by actin cytoskeleton staining (arrow in A and C). Nuclei were found homogenous in diameter and for DAPI staining intensity (B and C). Rarely, fragmented nucleus can be identified (arrowhead in B and C). THPTS-PDT treatment induced a reduction of filopodia (D, F) and many Rb cells exhibited heterogeneous nucleus aspect as seen with DAPI staining (E, F). Many fragmented nucleus (arrowheads in E and F) can be observed. Condensed genomic material was detected by the increased intensity for the DAPI signal (arrows in E and F). Many nuclei were found reduced in diameter compared to the control situation. Scale bar 30 µm.

Rb cells were incubated with low dose of THPTS (25 µg/ml) for 90 min to induce a moderate oxidative stress without massive cell death in order to visualize early events affecting the cell morphology compared to untreated cells. Rb cells were cultured in 10% lipumin (PAA Laboratories). This allowed a good adherence of the retinoblastoma cell lines on the glass surface, easing the immunocytochemistry staining process and analysis [23], [24]. We used DAPI nuclear marker to identify cell nucleus damage and phalloidin to stain the actin cytoskeleton and identify its fragmentation. In control condition (Figure 6), Rb cells showed a strong development of fillopodia as seen by actin cytoskeleton staining (arrow in figure 6A and C). Nuclei were found homogenous in diameter and exhibited similar DAPI staining intensity (Figure 6B and C). Still, sparsely, fragmented nucleus were identify in the culture (arrowhead in figure 6 B and C). After THPTS-PDT treatment, actin cytoskeleton was dramatically perturbed. Fillopodia were found mostly absent or drastically reduced in length (Figure 6D, F) and many Rb cells detached from the glass surface. Remaining cells exhibited a heterogeneous nucleus aspect as seen with DAPI staining. Many fragmented nucleus (arrowheads in Figure 6E and F) can be observed. Condensed genomic material was detected by the increased intensity for the DAPI signal (arrows in Figure 6E and F). Many nuclei were found reduced in diameter compared to the control situation. Chromatin condensations (pyknosis) preceding the nucleus fragmentations (karyorrhexis) are considered to be typical events in cells undergoing an apoptotic process.

**Figure 7:**
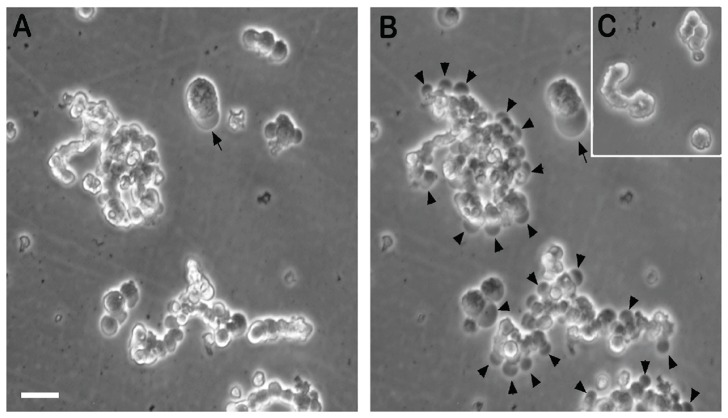
Membrane blebbing in treated cells. WERI-RB1 cells were incubated for 3 h with 100 µg/ml THPTS and bright field image were recorded every 30 s for 45 min. Start (A) and endpoints (B) are depicted, while endpoint of control culture without treatment is represented in insert (C). Few minutes after laser illumination, some cells already showed blebbing of the membrane (arrow in A). After 45 minutes (B) this feature was observed in many cells in culture (arrowheads) while absent of the control culture (C). THPTS-PDT treated cells showed therefore massive death by apoptosis. Scale bar in A, 15 µm.

Time lapse imaging was achieved on WERI-Rb1 cells treated with 100 µg/ml THPTS by recording bright field image every 30 seconds for 45 minutes after compound activation with proper laser illumination (Figure 7). Record started 15 minutes after laser stimulation; however some cells presented already a massive blebbing of their membrane (arrow in Figure 7A). After 45 minutes, those typical membrane damages can be observed in many cells in the field (arrowhead in Figure 7B), while completely absent of the untreated control cells culture after 45 minutes (insert Figure 7C). Membrane blebbing has been pointed out to be a characteristic feature of apoptosis while absent in necrotic process [29]. The time lapse of WERI-Rb1 (Video S1) confirmed the gene and immunocytochemistry data, demonstrating the rapid apoptotic death of Rb cells following THPTS –PDT.

### Subcellular distribution of THPTS

**Figure 8:**
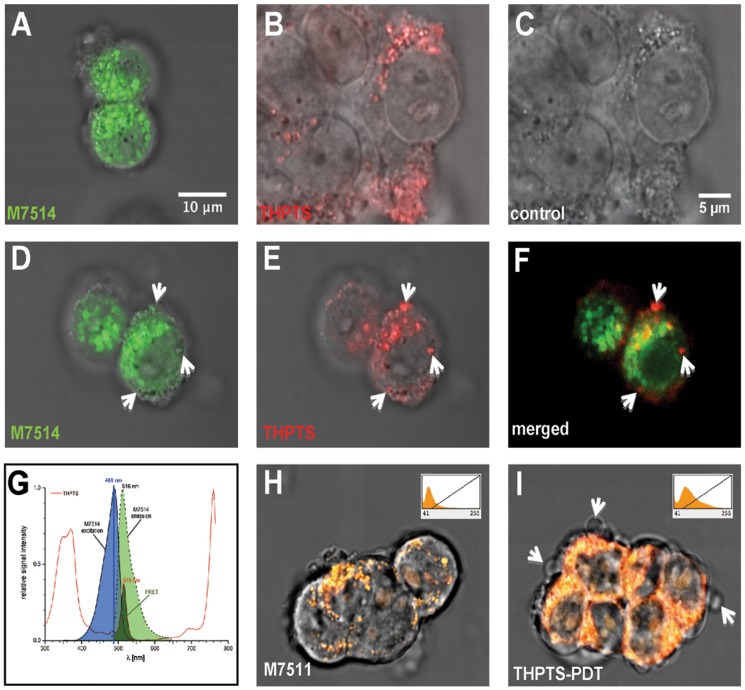
THPTS subcellular localization. MitoTracker green (M7514) original scan identifying mitochondria in Y79 cells (A) and resembling to subcellular THPTS distribution (B). (C) Control scan reveals no staining of THPTS at 488 nm excitation and detection with 650 nm long pass filter. (D-G) FRET pair identification in Y79 mitochondria as excited at 488 nm and observed at 505–530 nm (D) and above 650 nm (E). (F) Merged images revealed colocalization of M7514 and THPTS by double staining in numerous, though not all, mitochondria. Exclusive THPTS fluorescence distribution (arrows) account for a complete energy transfer from M7514 at those localizations while signal seen at 505–530 nm account for an excess of donor (D, F). M7514 and THPTS establish a FRET pair of fluorescence dyes as the emission maximum of MT7514 is 516 nm, which is the excitation maximum of THPTS; blue colored area highlights M7514 excitation curve; pale green color indicates M7514 emission spectrum and dark green area corresponds to the overlap with THPTS excitation spectrum (G). (H-I) Evidence for mitochondrial ROS production by THPTS excitation. Basic mitotracker M7511 fluorescence (H) was clearly enhanced following THPTS excitation at 514nm (I), indicating an oxidative process specifically due to THPTS-PDT (DAPI filter set, 5 min). Insets in indicate M7511 fluorescence intensity histogram (H-I). Scale bar in (A) applies to all images except for (B-C) where scale bar is shown in (C).

Incubation of Rb (Y79) cells with MitoTracker green (M7514) resulted in clear staining of mitochondria (Figure 8A). Using laser excitation at 514 nm, THPTS could be visualized with a 650 nm long pass filter and the fluorescence distribution closely resembled the M7514 labeling (Figure 8B), strongly accounting for a mitochondrial localization of THPTS. No signal was detected above 650 nm in absence of THPTS (Figure 8C). The pair of fluorescence dyes M7514 and THPTS established a Förster resonance energy transfer (FRET) system. Fluorescence emit by M7514 at 516 nm can excite THPTS that would then be detected above 650 nm (Figure 8G). Co-incubation of M7514 and THPTS revealed this FRET mechanism as THPTS was specifically detected in a subset of mitochondria after stimulation of mitotracker at 488 nm only (Figure 8D-F). The red THPTS-fluorescence not colocalized with green M7514 (arrows in Figure 8D-F) speaks in favor for a complete energy transfer from M7514 to THPTS, while remaining of M7514 fluorescence at 530–560 nm (Figure 8D,F) indicated that the donor dye was in excess. Therefore, part of the excitation energy was still released as photons. FRET pair indicated that THPTS was localized within a range of less than 10 nm to M7514 demonstrating that THPTS was indeed localized inside the Y79 mitochondria.

To further support this hypothesis we used the reduced mitochondrial marker M7511, which only emits fluorescence in its oxidized state. In absence of THPTS activation mitochondrial staining was weak, indicating only few oxidized M7511 species in the mitochondria (Figure 8H). However, the fluorescence was markedly increased in intensity and showed wider distribution following THPTS-PDT with UV-light using the DAPI filter set of the microscope (Figure 8I). After 5 min of illumination, first membrane blebbing occurred, indicating apoptosis (arrows in Figure 8I).

## Discussion

Here we identified the potential tumoricidal effect of THPTS on two human retinoblastoma cell lines. While exhibiting no cytotoxic effect per se, 200 µg/ml THPS was able to induce a complete cell death once activated with a 760 nm laser beam at 60 J/cm^2^ and 100 mW/cm^2^. The maximal cytotoxic effect was reached even with incubation time as short as 1.5 hours. In addition there are clear dose-dependent and an incubation length–dependent effects, meaning that the same cytotoxic effects can be reached with lower THPTS concentrations by increasing the incubation time. This is of major interest to obtain the best ratio for cancer cell death vs preservation of the surrounding normal cells. We were indeed able to demonstrate the high specificity of the compound for cancer cells as normal RPE cells were significantly (50 µg/ml: p<0.05; 100 µg/ml: p<0.01; 200 µg/ml: p<0.001) less affected by THPTS-PDT treatment. For instance their survival after 3 h is 3 fold higher as for retinoblastoma using 100 µg/ml of THPTS (Figure 3). These results were in accordance with previous publication that reported a high specificity of the compound for various tumor cell types with a high TNTR ranging from 8 to 50 [17]. It has to be noted that primary RPE cells in vitro do proliferate while this does not occurs normally in vivo. Proliferating RPE cell in vitro might be more sensitive to THPTS than RPE cell in situ due to an increased metabolism that may facilitate the entry of THPTS in the cell. The molecular mechanism underlying the target effect of THPTS-PDT is still largely unknown but might be due to the well documented increase in metabolic uptake occurring in tumor cells [30]. THPTS would then accumulate faster in cancer cells. Also, the resting membrane potential of many cancer cells is hyperpolarized compared to normal cells [31] and may therefore facilitate the uptake of the positively charged THPTS present in the medium as well as its retention in the cancer cell. Last but not least, the mitochondrial hyperpolarization occurring in tumor cells [32] may allow THPTS to target more specifically this subcellular compartment leading to the high efficiency of treatment. Functional mitochondria indeed are essential to cancer cell development and highly sensitive to oxidative stress [33]. They are actually considered as the Achilles heel of cancer cells and target for tumor elimination using mitochondriotoxic compounds [34], [35]. To further analyse the mechanism of cell death induced by THPTS-PDT in retinoblastoma we studied the quantitative expression for mRNA of genes related to apoptosis (hATF3; hJUN; hHSP70B) or stressful growth arrest (hp27) including DNA damage (hGADD45G; hGADD153) and other severe stress (hHSP105) [26]. Both retinoblastoma cell lines tested, exhibited a massive upregulation of these genes. ATF3 encodes a pro-apoptotic protein that mediates cytotoxic agents (i.e. ROS) induced apoptosis in various systems [36]. hJUN in combination with c-Fos, forms the AP-1 early response transcription factor highly sensitive to ROS. Rapid and strong upregulation of ATF3 and hJUN mRNAs suggest an apoptotic cell death mechanism induced by THPTS-PDT. Moreover, overexpression of hHSP70B indicates more precisely a Fas-dependent apoptotic mechanism [27]. Also, increased levels of the mRNA for p27Kip1 protein typically cause cells to arrest in the G1 phase of the cell cycle further driving apoptotic cell death [37]. GADD45G increase reflects the stressful growth arrest conditions via treatment with DNA-damaging agents [38]. Increased GADD153 also participate to the arrested cell growth and promote programmed cell death, in part via activation of AP1 target gene [39]. Last but not least, it has been shown that cytosolic heat shock proteins such as HSP105 are preferentially induced in response to specific and localized intramitochondrial damage [40]. The induction of apoptosis was also suggested by the chromatin condensation (pyknotic nucleus) and the karyorrhexis (nucleus fragmentation) seen in Rb cells treated with mild THPTS-PDT (25 µg/ml, 90 min). Together with cytoskeletal restructuration and membrane blebbing, those damaged nuclei are hallmarks of the undergoing apoptotic process. Altogether, our results demonstrate that THPTS-PDT leads to a massive cell death via apoptotic pathway induction in response to oxidative stress targeting the mitochondrial compartment. This cytotoxic effect seems to be specific for cancer cells while sparing the normal RPE cells to a great extend.

### Advantage of PDT over other cancer therapies

Most of the commonly used cancer therapies are highly immunosuppressive while PDT produces an acute activation of the immune system that attracts leukocytes to treated tumors [18]. PDT might therefore increase the immunogenicity of dead tumor cells by exposing or creating new antigens [41] and by inducing heat-shock proteins that increase the efficiency of antigen cross-presentation to form more effective tumor-specific cytotoxic T cells [42]. In addition, the pro-inflammatory effects of PDT might increase dendritic-cell migration, antigen uptake and maturation leading to adaptive immunity [43]. PDT can promote tumor cures and long-lasting tumor-specific memory [44], as has been shown by the rejection of tumors on rechallenged rodent models [45]–[48]. Mechanism of action involves neutrophil cells infiltration and activation of natural killer cells that are both essential for the generation of tumor-specific primary and memory CD8 (+) T-cell responses [43], [49].

Like PDT, chemotherapy is based on the preferential uptake of the compound by cancer cells thus leading to increased cytotoxity in tumoral vs. healthy cells. However, contrary to photosensitizers, chemotherapy involves compounds that are cytotoxic per se. The tumor cell can develop an acquired resistance mechanism that will reduce the efficiency of the treatment and leave the organism vulnerable to relapse [50]. This is very unlikely to happen in PDT as photosensitizers are not cytotoxic per se but rather induce oxidative stress in the target cell via ROS. The activation of the immune system combined with the low resistance mechanism make PDT an ideal cancer curative treatment. However, many technical limitations are linked to the dozen of clinically approved photosensitizers and limit their use to few applications, mainly in dermatology, urology and gastroenterology [51], [52]. In neuro-ophthalmology, the only clinical application for PDT is the neovascularization associated with macular degeneration [53]. However the actual development of a new generation of photosensitizers should definitely lead to preclinical evaluation of PDT as a therapeutic option for the treatment of retinoblastoma [54]. Its use in the handling of retinoblastoma may consist in an promissing therapeutic alternative not only in Western countries in case of resistant tumor forms but also in less advanced countries due to the reduced logistic and associated costs of the PDT treatment.

### Advantage of THPTS compared to other photosensitizers

A main actual limitation of PDT is that visible light is needed to activate most of the clinically approved photosensitizers. Visible light cannot pass through more than 10 mm of tissue. For this reason, PDT is usually used to treat tumors on or just under the skin or on the lining of internal organs or cavities [55]. Therefore, actual PDT is inefficient for treating large, pigmented or deep tumors. In addition, high amounts of energy needed to activate actual compounds lead to skin lesions and burns [51]. THPTS is activated by infrared wavelength, thus limiting damages to normal tissue while it increases the therapy depth up to 25 mm. Another limitation is the rapid turnover (within minutes) of some compounds that requires performing PDT immediately after incubation with the photosensitizer [56]. Here we demonstrate that THPTS rapidly entered the cells, as the maximal effect is reach after 1 to 3 hours for all tested concentrations and remained active over a long period with a half life in the range of a day, while skin photosensibility can last for months with approved photosensitizers (European Medicine Agency requirement WC500024398). THPTS compound is positively charged and therefore may target more specifically the mitochondrial compartment leading to faster and targeted oxydative damage [22], [57]. Last but not least, TNTR of THPTS is higher than many other commercially available photosensitizers [22].

### Future development

Next to in vitro study, the proof of concept for the use of THPTS in the handling of retinoblastoma requires in vivo experiments in which a tumor would develop among normal surrounding retinal cells. The cohabitation of normal and tumoral retina cells is essential to demonstrate the high specificity of THPTS for cancer cells. There are currently two types of rodent models for proper retinoblastoma study in vivo: xenograft and transgenic mouse [58]. Injection of human retinoblastoma in immunocompetent newborn rats recapitulates the developmental environment of the human retinoblastoma [59], [60]; while in a LH beta -Tag transgenic mouse, a highly expressed transgene drives the overexpression of the SV40 large T antigen leading to bilateral retinal tumor development that resembles human retinoblastoma, occupying approximately a quarter of the retinal area at 10 weeks of age [61], [62]. Both models are relevant for retinoblastoma study because the tumors are developing in situ, within the retinal tissue, in immune competent animals. Outcomes obtained with THPTS may not only be beneficial for retinoblastoma handling but also for various CNS tumors in which preservation of surrounding tissue is critical. Among those, glioblastoma are highly aggressive brain tumors with little or no animal model available [63]. Interestingly, some subtypes of glioblastoma share common genetic abnormalities with retinoblastomas as mutations in the RB1 specific pathway are associated with the shortest survival in patients affected by glioblastoma [64]–[66]. Thanks to the accessibility of the retinal tissue and the many non-invasive methods of analysis available: scanning laser ophthalmoscope, optical coherence tomography and electroretinogramm to name a few, THPTS-PDT on retinoblastoma in-vivo may also consist in a useful model for glioblastoma studies

## Supporting Information

Video S1 **Supplementary data.** Time lapse movie of membrane blebbing in WERI-RB1 cells. RB1 were incubated for 3 h with 100 µg/ml. Recording started 15 min after the irradiation and THPTS and bright field image were recorded every 30 seconds. Arrowheads indicated the blebbing spots an hour after the irradiation. The massive blebbing suggests that THPTS-PDT treated cells were dying by apoptosis.
(WMV)
